# Improved osteoblast function on titanium implant surfaces coated with nanocomposite Apatite–Wollastonite–Chitosan– an experimental in-vitro study

**DOI:** 10.1007/s10856-022-06651-w

**Published:** 2022-02-21

**Authors:** Shayanti Mukherjee, Smriti Sharma, Vivek Soni, Amruta Joshi, Amit Gaikwad, Jayesh Bellare, Jyoti Kode

**Affiliations:** 1 Kode Lab, Tumor Immunology & Immunotherapy Group, Advanced Centre for Treatment, Research & Education in Cancer, Tata Memorial Centre, Kharghar, Navi Mumbai, 410210 India; 2 The Ritchie Centre, Hudson Institute of Medical Research, Clayton, VIC 3168 Australia; 3 Department of Obstetrics and Gynaecology, Monash Medical Centre, Monash University, Clayton, VIC 3168 Australia; 4 Department of Chemical Engineering, School of Biosciences and Bioengineering, Indian Institute of Technology Bombay, Powai, Mumbai, 400076 India; 5 Department of Chemical Engineering and Wadhwani Research Center for Bioengineering, IIT-Bombay, Mumbai, 400076 India; 6 Deptartment of Orthodontics, D.Y. Patil University, School of Dentistry, Navi Mumbai, India; 7 Department of Periodontics, MGM Dental College and Hospital, Navi Mumbai, 410209 India; 8 Department of Prosthodontics, MGM Dental College and Hospital, Navi Mumbai, 410209 India; 9 Department of Chemical Engineering and Wadhwani Research Center for Bioengineering, IIT-Bombay, Powai, Mumbai, 400076 India; 10 Homi Bhabha National Institute (HBNI), Training School Complex, Anushakti Nagar, Mumbai, 400094 India; 11 Present Address: Principal Scientist I at Roche Sequencing Unit, Pleasanton, CA 94588 USA

**Keywords:** Biomimetic material, Dental/Orthopedic implant, Osseointegration, Osteoblast, Titanium, Systematic in vitro approach

## Abstract

**Background:**

There is a continuous research in the area of biomimetic coatings on the titanium (Ti) implant surfaces for improved survival and long-term successful outcomes in the field of dentistry and orthopedics. In-vitro approaches are ideal systems for studying cell-material interactions without complexity and interference observed in in-vivo models.

**Purpose:**

The present study was undertaken to evaluate the osteoblast characteristics and function on Ti substrates coated with the novel composite coating of ceramic apatite-wollastonite (AW) and polymer chitosan.

**Materials and methods:**

Ti substrate coated with composite AW-Chitosan was synthesized, using electrophoretic deposition. MG-63 cells were seeded onto the coated substrates and cellular morphology and growth was assessed using Scanning Electron Microscopy (SEM) and Laser Scanning Microscopy (LSM). Osteocalcin expression of the seeded cells was assessed by FITC tagging and LSM analysis. Alizarin Red S staining and Confocal LSM (CSLM) analysis was used to study the in-vitro mineralization on the titanium samples.

**Results:**

The AW-Chitosan coating on Ti samples by electrophoretic deposition exerted significant positive influence on cell proliferation, growth and mineralization as compared to uncoated titanium samples. Scanning electron microscopy and laser confocal microscopy experiments revealed that the coating was non-toxic to cells, enhanced adhesion and proliferation of MG-63 cells. Increased functional activity was observed by increased production of bone-specific protein osteocalcin and mineralized calcium through day 7 and 14.

**Conclusions:**

The present study underscores that optimal inorganic-organic phase nanocomposite crack-free coating created on Ti by simple, cost-effective electrophoretic deposition technique may have osteoconductive potential and may have wide application in the field of implantology.

**Figure:**
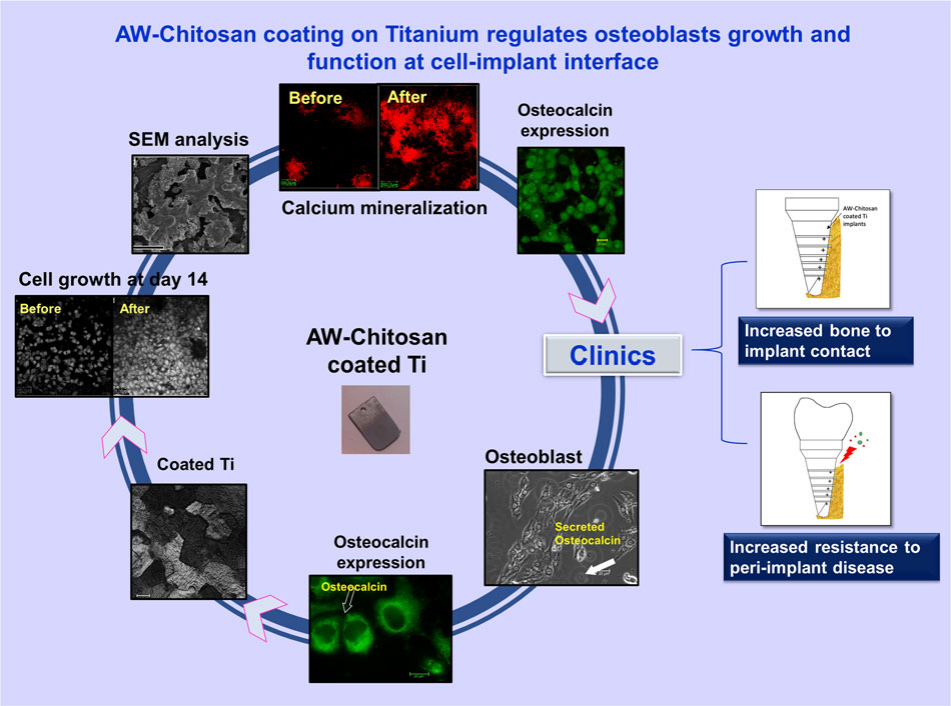
Graphical abstract

## Introduction

Titanium (Ti) and Ti alloys are widely used in the field of implantology on account of their good mechanical properties and biocompatibility [1, 2]. The success and failure of an implant are largely dependent on the extent to which it integrates into the surrounding bone. The greater is the osteointegration, the higher is the initial mechanical stability and lesser is the probability of implant loosening with formation of fibrous tissue at the interface and implant failure. Hydroxyapatite (HA) coatings have already evoked much interest as it has possibility of achieving a real chemical bond between bone tissue and implant surface with absence of fibrous tissue at the interface [3]. However, long term stability of the interface between ceramic coating and metal, as well as possibility of HA resorption with subsequent loss of integrity of the coating layer, could represent a serious problem. Since the most appropriate surface coating has not yet been identified, new coating techniques with physicochemical and morphological modifications are continuously being developed and investigated. Organic–inorganic composites, prepared from bone-bonding bioactive ceramics and biopolymers, are useful for novel bone substitutes having mechanical properties analogous to natural bone [4]. Natural bone has a three-dimensionally woven apatite-polymer structure that is constructed from inorganic apatite crystals and organic collagen fibers and exhibits specific mechanical properties, such as high fracture toughness and flexibility.

The bioactive ceramics viz. Bioglass [5] sintered HA [6] and glass-ceramic Apatite-wollastonite (AW) containing crystalline apatite and wollastonite [7] have been used for bone repair in clinical fields. Their bone-bonding ability of bioactive ceramics makes a tight fixation between bone and artificial materials. However, bioactive ceramics, known so far, have limitation on clinical application, because of their mechanical properties such as high Young’s modulus, low toughness, and brittle character [4].

In order to circumvent this problem, organic–inorganic composite consisting of bioactive ceramics and organic polymers have attracted much attention as novel bioactive materials. Composites consisting of biodegradable polymer and hydroxyapatite have been researched by investigators due to their composition and structure analogous to those of natural bone Some biodegradable polymers viz. collagen [8], gelatin [9] and chitosan [10] have been used along with HA in recent years in composite preparation. Usage of organic polymers with high chemical durability is expected to give bioactive composites the ability of retention of mechanical strength for a long period after implantation in the body. However, the low solubility of the biopolymer in conventional organic solvents has been a major hurdle to explore its use in composite coatings for healing bone defect.

Sharma et al. have successfully demonstrated biopolymer chitosan reinforcement in AW composite coating on Ti substrates by electrophoretic deposition (EPD) technique [11, 12]. This coating could provide excellent in vitro bioactivity with enhanced mechanical properties in simulated body fluid [11] as compared to uncoated Ti substrates. Further, they also demonstrated in vivo ability of these coated Ti substrates to heal tibial bone defects in rabbits compared to uncoated one. The present study was undertaken to evaluate in vitro the osteoconductivity of composite coated on Ti substrates and their suitability as substrate for supporting the proliferation and function of human osteoblasts. Expression and localization of osteocalcin within cells and its secretion in surrounding medium was considered as marker of osteoblast differentiation and mineralization.

## Materials and methods

The present study was an in-vitro study performed using MG-63 established human osteoblast cell line procured from NCCS, Pune, India. Ethical approval was not required for the study, as the study did not involve the use of any human or animal subjects or primary culture cells. CRIS (Checklist for Reporting In-vitro Studies) guidelines were followed during the formulation and execution of the present in-vitro study [13].

### Preparation of Ti substrate and AW-Chitosan coating

AW powder formed by sol-gel route [14] was used in this study for synthesizing the composite coating. Chitosan was obtained from Otto chemicals (98% deacetylation). Ti sheets (*n* = 40) of dimension (10 mm × 15 mm × 0.5 mm) with measured purity of 98% using EDAX (Quanta 200, FEI) were used as the test substrate. Titanium substrates coated with composite AW-Chitosan were synthesized, using electrophoretic deposition as described by Sharma et al. [11, 12]. Uncoated titanium substrates were used as control. Each experiment employed Ti implant sheets in duplicate per parameter. Each experiment was repeated three times for the purpose of accuracy validation and reproducibility.

### Cell Culture and osteocalcin localization in MG63 cells

MG-63 osteoblast-like cells (human osteosarcoma cell line) were cultured in Dulbecco’s modified eagle’s medium (DMEM) containing 2 mM L-glutamine, supplemented with 10% fetal calf serum and 1% antibiotic solution at 37 °C in a humidified atmosphere of 5% CO_2_. MG-63 cells were plated onto coverslips for assessing osteocalcin localization within the cells. After sufficient growth, the medium was discarded, and each coverslip with adherent cells was washed once with PBS, fixed with 4% formaldehyde in PBS for 5 min, and washed with PBS once again. The cell seeded coverslips were rinsed with PBS, permeabilized in 0.3% (v/v) in Triton X-100 for 90 s and fixed in chilled methanol at −20 °C for 10 min. To measure the formation of extracellular matrix (ECM) and cell growth, non-specific sites were blocked with 10% normal goat serum, then incubated for 1 h at 37 °C with the rabbit polyclonal antibody against human Osteocalcin (Santacruz Biotechnology, USA) as primary antibody and secondary antibody tagged with FITC (Sigma, USA). The coverslips without primary antibody served as negative control. The stained samples were observed under Laser Confocal Microscope LSM 510META (Carl Zeiss, Germany).

### MG-63 osteoblast cell seeding on Ti substrates

The AW-Chitosan nanocomposite coated/uncoated titanium implants were sterilized by gamma irradiation at 20 K Gy 30 °C in Gamma Chamber (GC-1200 having ^60^Co as the source) at Tata Memorial Hospital, Parel, Mumbai. The radiation dose given was according to the standards of the International Atomic Energy Agency (IAEA). Ti discs were placed in 60 mm petri dishes under sterile conditions and equilibrated with complete medium. The cells were plated onto the discs according to the protocol published earlier [15]. Briefly the cells were plated at a density of 5 × 10^5^ cells/disc in 60 mm petri dishes, in a 25 μl of medium. After initial attachment for two hours at 37 °C, 5 ml of growth medium was added. The cell/discs constructs were then cultured in a humidified atmosphere at 37 °C with 5% (v/v) CO_2_ for 7 and 14 days. The medium was changed every second day. After incubation each disc was carefully washed with normal saline and treated with 3% Glutaraldehyde (EM grade) for 2 h at room temperature. Cell/discs were stored in PBS at 4 ^o^C till further use. 10 Ti discs were used per coated/uncoated set and Ti discs were randomized using computer generated random sequencing method prior to their use before using for individual read out assay to determine accuracy of test employed. Blinding approach was followed while acquiring and analyzing data.

### Cell morphology, adhesion and proliferation

The discs were dehydrated in grades of alcohol and then sputter coated with gold alloy, and viewed using SEM at 10 kV. Cells stained for osteocalcin were also studied for their morphology and growth by Laser scanning microscopy on LSM 510 META (Carl Zeiss, Germany).

### Cell metabolic activity

Ti discs bare and coated with nanocomposite were kept overnight in complete medium in tissue culture wells. This served as leached media from Ti discs. MG-63 5 × 10^3^ cells were cultured in 96-well flat bottom microtiter plate. Ti discs leached media were added at 50% and 100% and cells were further incubated for another 24 h at 37 °C incubator in humidified atmosphere with 5% CO2 in air. A total of 10% of cell CCK-8 dye was added to each well and incubated for 4 h in the dark at 37 °C humidified incubator with 5% CO_2_. Thereafter, the medium was collected and its absorbance was measured at 450 nm in a Microplate Reader (Biotek FL-600, Bio-Tek, Winooski, VT). Medium with only cells and plain medium without cells were used as controls.

### Osteocalcin expression on coated Ti substrates

Intracellular and secreted osteocalcin was visualized in MG 63 cells as described previously [16]. Briefly, the discs plated with cells were rinsed with PBS, permeabilized in 0.3% (v/v) in Triton X-100 for 90 seconds and fixed in chilled methanol at −20 °C for 10 min. To measure the formation of ECM and cell growth, non-specific sites were blocked with 5% bovine serum (BSA, Sigma), then incubated for 1 h at 37 °C with the mouse monoclonal antibody against human Osteocalcin (dilution 1:100, RnD Biosciences) as primary antibody and secondary antibody tagged with fluorescein isothiocyanate (FITC) (Sigma, MO). Images of the resultant fluorescence were then collected on Laser Confocal Microscope LSM 510META (Carl Zeiss, Germany). The images were analysed and compared using LSM 510 Imaging software, Carl Zeiss, Germany.

### In-vitro mineralization

Composite coated substrates seeded with MG-63 cells were fixed in 2.5% glutaraldehyde for 2 h at room temperature and stored under PBS buffer at 4 °C for detection of mineralized calcium at seven and 14 days. Calcium precipitates were detected after fixation 2.5% glutaraldehyde for 2 h and followed by staining with Alizarin Red S (40 mM, pH 4.2) for 10 min. Fluorescence images were visualized and captured on Laser Confocal Microscope (Confocal Microscope- Olympus-FV 500).

### Statistical Analysis

Results were expressed as Mean ± Standard Error for CCK-8 dye assay. Comparison of absorbance of cells in different groups done using Student’s *t* Test. All *p* values were two tailed and a level of <0.05 was accepted as statistically significant. Analysis of images captured on fluorescent microscope for inverted phase contrast transmission image and green-labelled fluorescent osteocalcin images, analysis was done by dedicated specialized software provided with Laser confocal microscope. Alizarin red stained red images also were acquired on laser confocal microscope and images were processed and analyzed for histogram and intensity values using dedicated software. Image data was also processed using ImageJ software of NIH, USA.

## Results

### Osteocalcin localization in MG-63 cells

**Fig. 1:**
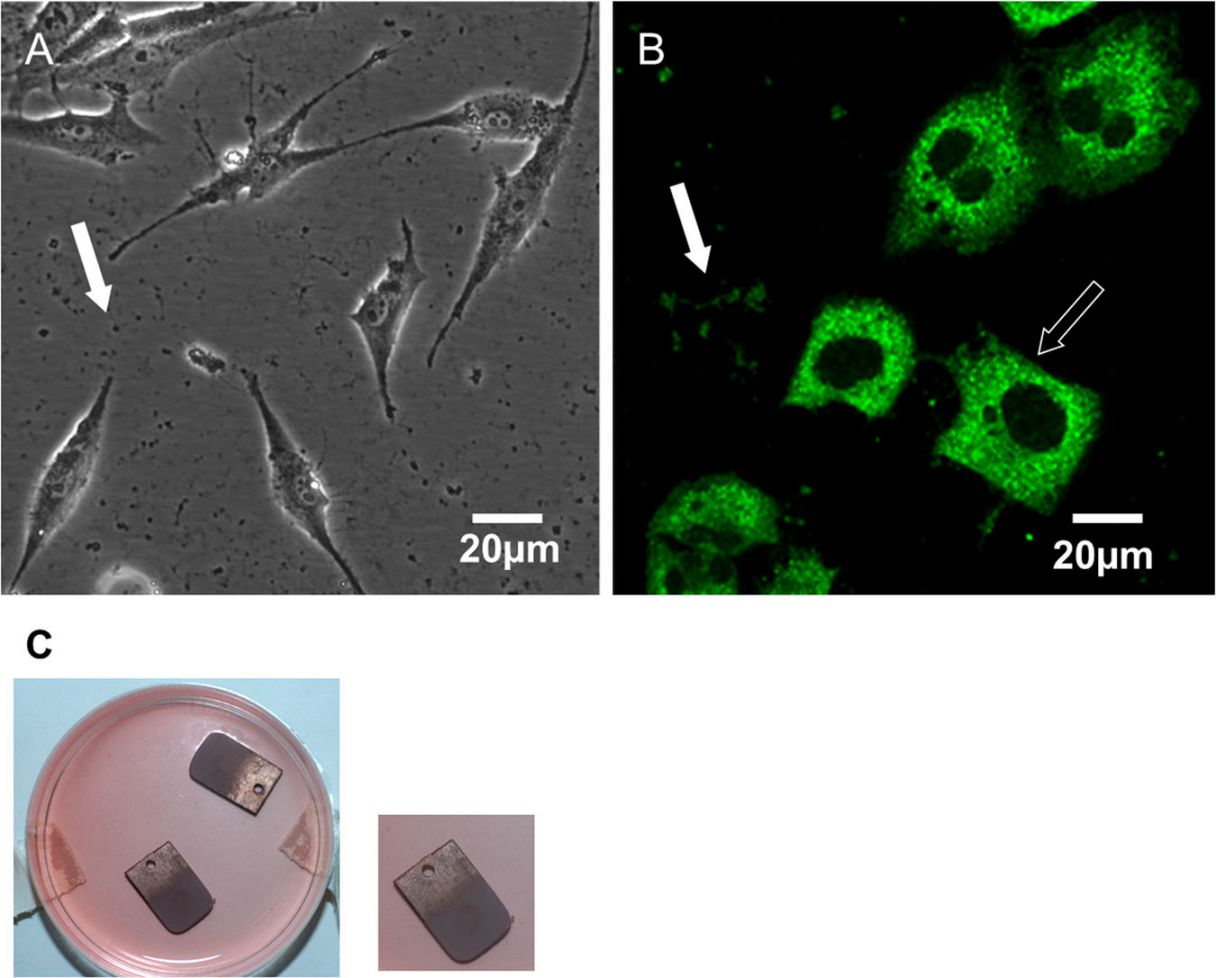
Panel (**A**) represents bright field microscopy image of MG-63 cell culture. White arrow indicates secreted Osteocalcin molecules. Panel (**B**) shows Osteocalcin expression in perinuclear region within MG-63 cells (indicated by hollow arrow) and secreted osteocalcin (indicated by solid arrow) after staining with anti- human Osteocalcin antibody and secondary antibody conjugated to FITC. Image was captured under laser confocal microscope (Scale bar = 20 micron). Panel (**C**) shows MG-63 culture on coated/uncoated Titanium samples in 60 mm petridish

MG-63 cells grown on coverslip are shown in Fig. 1A. This culture routinely demonstrated presence of secreted osteocalcin, observed as black spots in culture plates/coverslips (indicated with a white solid arrow). Immunofluorescence experiment demonstrated that almost all MG-63 cells expressed intracellular osteocalcin (Fig. 1B, Supplementary Video 1). Osteocalcin was found to be located within cytoplasm with increased concentration at perinuclear spaces (Fig. 1B). Staining of secreted osteocalcin (indicated as white solid arrow) was prominent every time the culture was stained. This data is well corroborated by flow cytometry which showed 99% cells expressed intracellular osteocalcin (Supplementary Material 1A). Reverse-transcriptase-PCR of MG-63 cells demonstrated significant expression of transcripts for bone-specific transcription factor cbfa-1 and osteocalcin (Supplementary Material 1B).

### MG-63 cell morphology, adhesion and proliferation

**Fig. 2:**
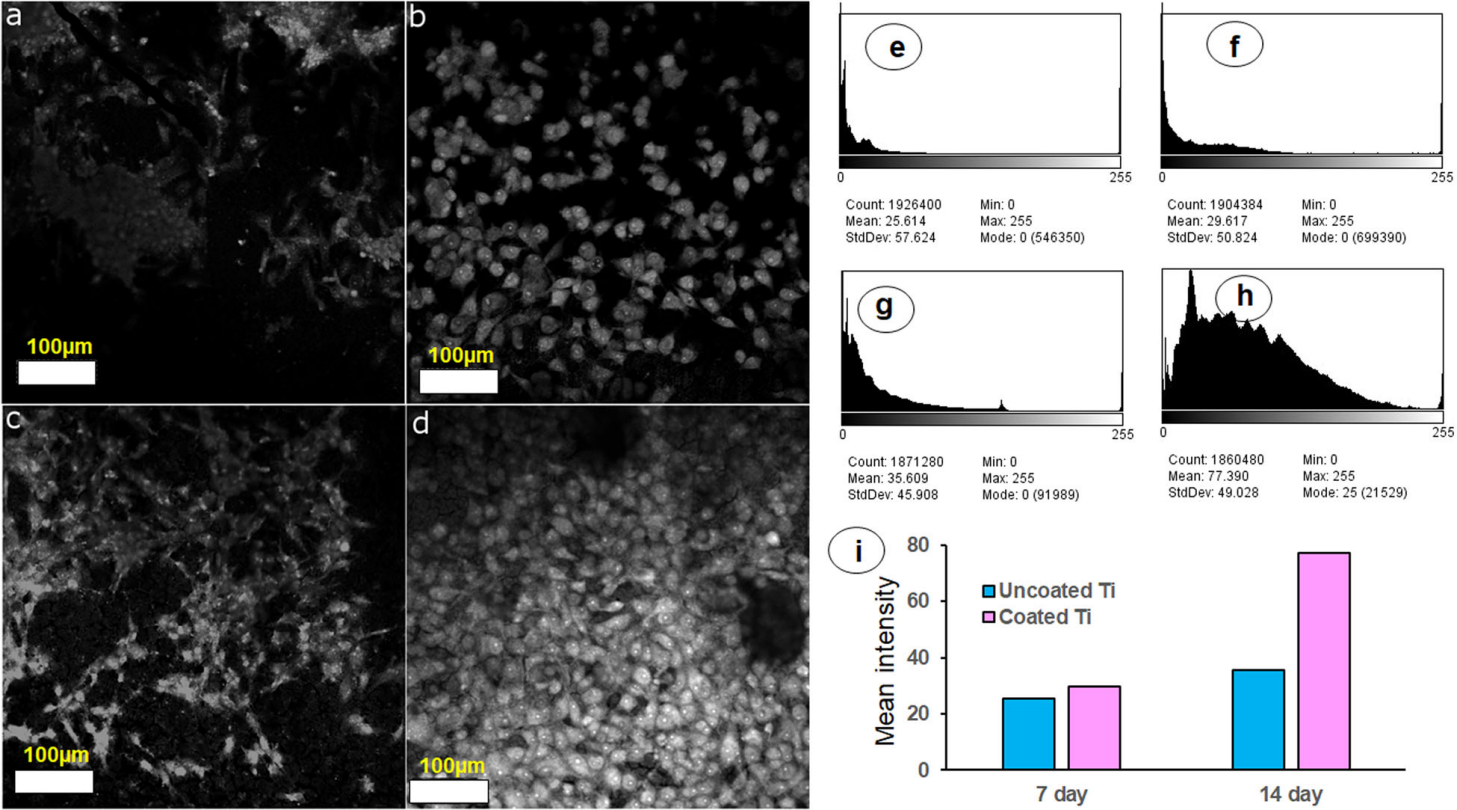
Representative reflection images of MG-63 seeded on uncoated Titanium samples at (**a**) seven days, (**b**) 14 days and coated Titanium samples at (**c**) 7 days, (**d**) 14 days. Images were captured using Laser Confocal microscope (Scale Bar = 100 micron). Respective histograms were computed using Image J software (NIH, USA). Uncoated Ti (panels **e** and **f**), Coated Ti (panels **g** and **h**); seven day cell growth (panels **e** and **g**) and 14 day cell growth (panels **f** and **h**). Mean intensity computed from histograms was plotted as bar graph in panel **i**

The culture system where MG-63 cells were seeded on Ti substrates and cultured upto 14 days is shown in Fig. 1C. The coated titanium sheet after cell culture was captured on LCM. Criss-cross pattern of organized layer coating was observed on titanium substrate. (Supplementary material 2). Confocal microscopy was employed to study cell proliferation and growth on uncoated (control) and coated titanium substrates. Cell growth, adhesion and morphology is evident in grey image of cells on Ti substrates. Micrographs in Fig. 2 exhibit that coated titanium substrate supported better MG-63 cell attachment and growth compared to the uncoated substrate. The cell number increased from day 7 to 14. Few cells were flattened in uncoated substrate while almost all cells attained fibroblast phase using coating as support extracellular matrix (ECM) on coated Ti substrate. Area covered by the cells in terms of mean grey intensity was quantified using Image J software (http://rsbweb.nih.gov/ij/) to assess indirectly the cell density on the substrate. For uncoated substrate, mean intensity of grey field at 25.6 (measured at seven days) covered by the cells increased up to 29.6 at 14 days (Fig. 2 e, f and i). In case of coated substrate, mean intensity of grey field at 35.6 (measured at seven days) increased up to more than 77 at 14 days (Fig. 2 g, h and i). This suggests that AW-Chitosan coating was nontoxic to cell growth and is more conducive for osteoblastic cell growth compared with uncoated one.

**Fig. 3:**
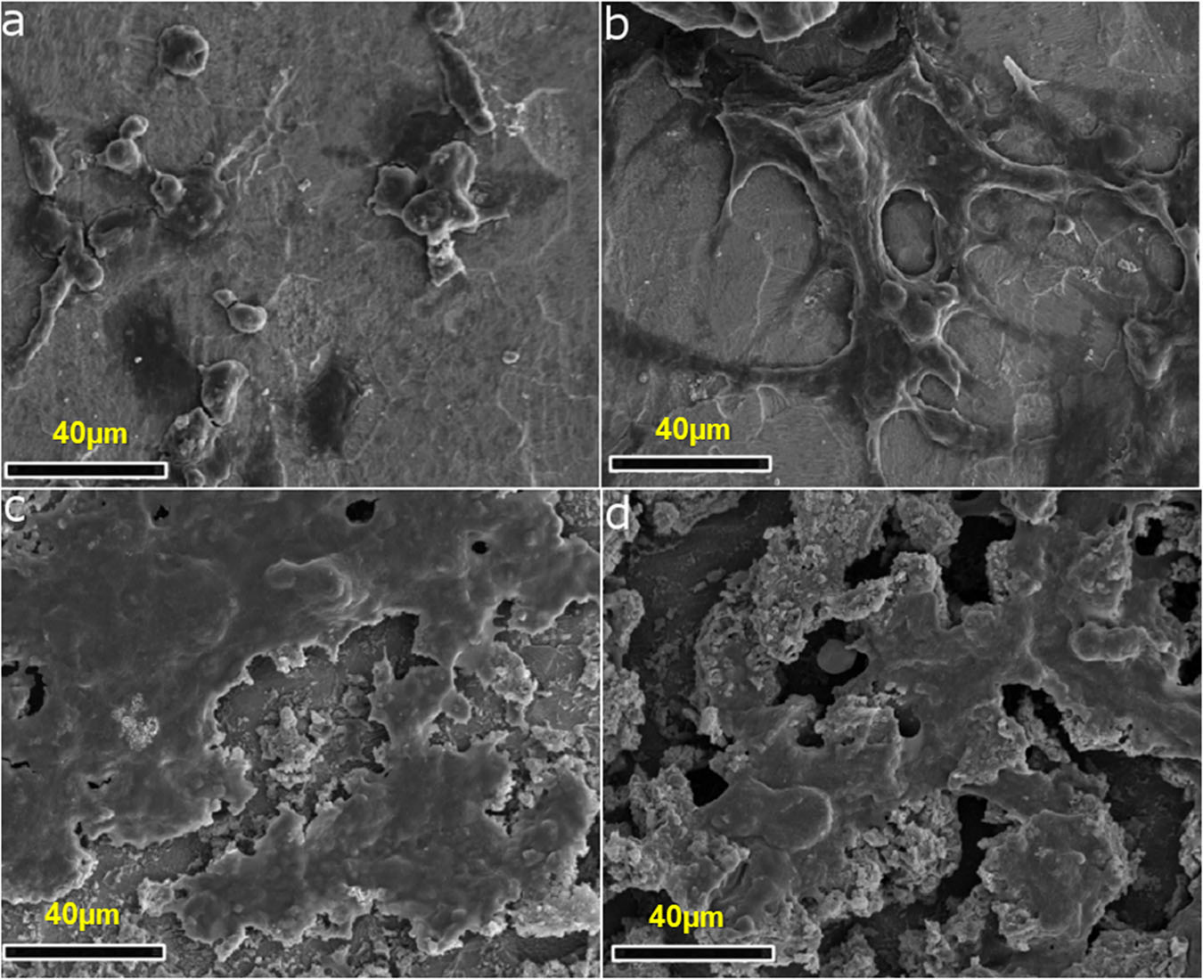
Representative SEM micrographs of MG-63 seeded on uncoated Titanium samples at (**a**) 7 days, (**b**) 14 days and coated Titanium samples at (**c**) 7 days, (**d**) 14 days and (Scale Bar = 40 micron)

To investigate detailed morphological and substrate surface features, scanning electron microscopy was performed on uncoated and coated Ti substrates (Fig. 3). Figure 3a shows that very few MG-63 cells attached on the surface of uncoated titanium substrate at 7 days and their shape was more spherical indicating lack of cell-matrix interaction. At 14 days, cells developed flattened morphology and secreted ECM to some extent (Fig. 3b). As against this, composite layer on coated Ti substrate supported cell-matrix interaction. At 7th day, cells grew in the form of flattened sheet (Fig. 3c). At 14 days, cells formed thick layer of ECM which completely covered the substrate surface (Fig. 3d). These findings from SEM studies corroborate the confocal microscopy data on morphology and cell adhesion (Figs. 2 and 3).

MG-63 cells were cultured for 24 h in leached culture media of Ti bare and Ti-coated with nanocomposite. There was negligible effect on cell metabolic activity in Ti uncoated- leached media at both dilutions (Ti50 and Ti100) as compared to control media (Supplemental Material S2). While leached media from Ti coated with nanocomposite (TiCo50 and TiCo100) showed marginal decrease in metabolic activity which could be associated with initiation of mineralized matrix formation (Supplemental Material S3).

### In-vitro mineralization

**Fig. 4:**
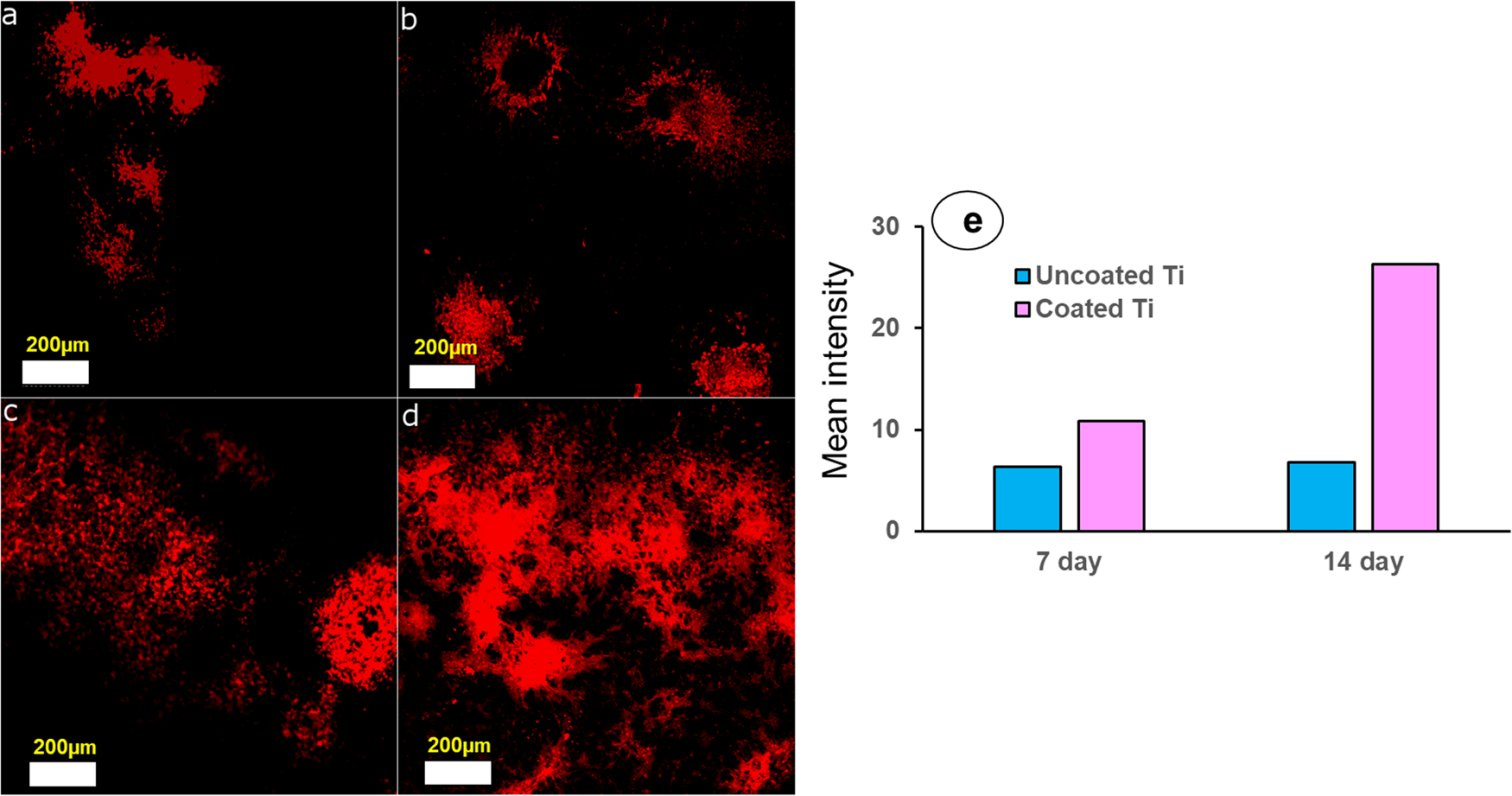
Representative images of calcium mineralization after alizarin-red S staining of MG-63 seeded on uncoated Titanium samples at (**a**) 7 days, (**b**) 14 days and coated Titanium samples at (**c**) 7 days, (**d**) 14 days (Scale Bar = 200 micron). Images were captured on Laser Confocal microscope using emission filter BP 560–615. Respective histograms were computed using Image J software (NIH, USA). Mean red color intensity obtained from histograms was plotted as bar graph in panel (**e**)

Alizarin red S staining was used to evaluate calcium-rich deposits formed by cells in culture. Image of mineralized calcium deposits in trabecular bone section under Laser Confocal microscope is given as supplementary material 4. For both uncoated and composite coated titanium substrates, mineralization was found to be increased from 7 to 14 days (Fig. 4). Also, the mineralization was observed comparatively more for coated substrates at both the time intervals compared with uncoated substrate. Area of mineralization was quantified in terms of mean red color intensity using Image J software. For uncoated substrate, mean intensity of red color field at 6.3 (measured at seven days) covered by the cells increased up to 6.7 at 14 days (Fig. 4 e). In case of coated substrate, mean intensity of red color field at 10.9 (measured at seven days) increased up to more than 26 at 14 days (Fig. 4 e). This result suggests that coated substrate resulted in higher mineralization as compared to uncoated substrate.

### Osteocalcin expression in-vitro

**Fig. 5:**
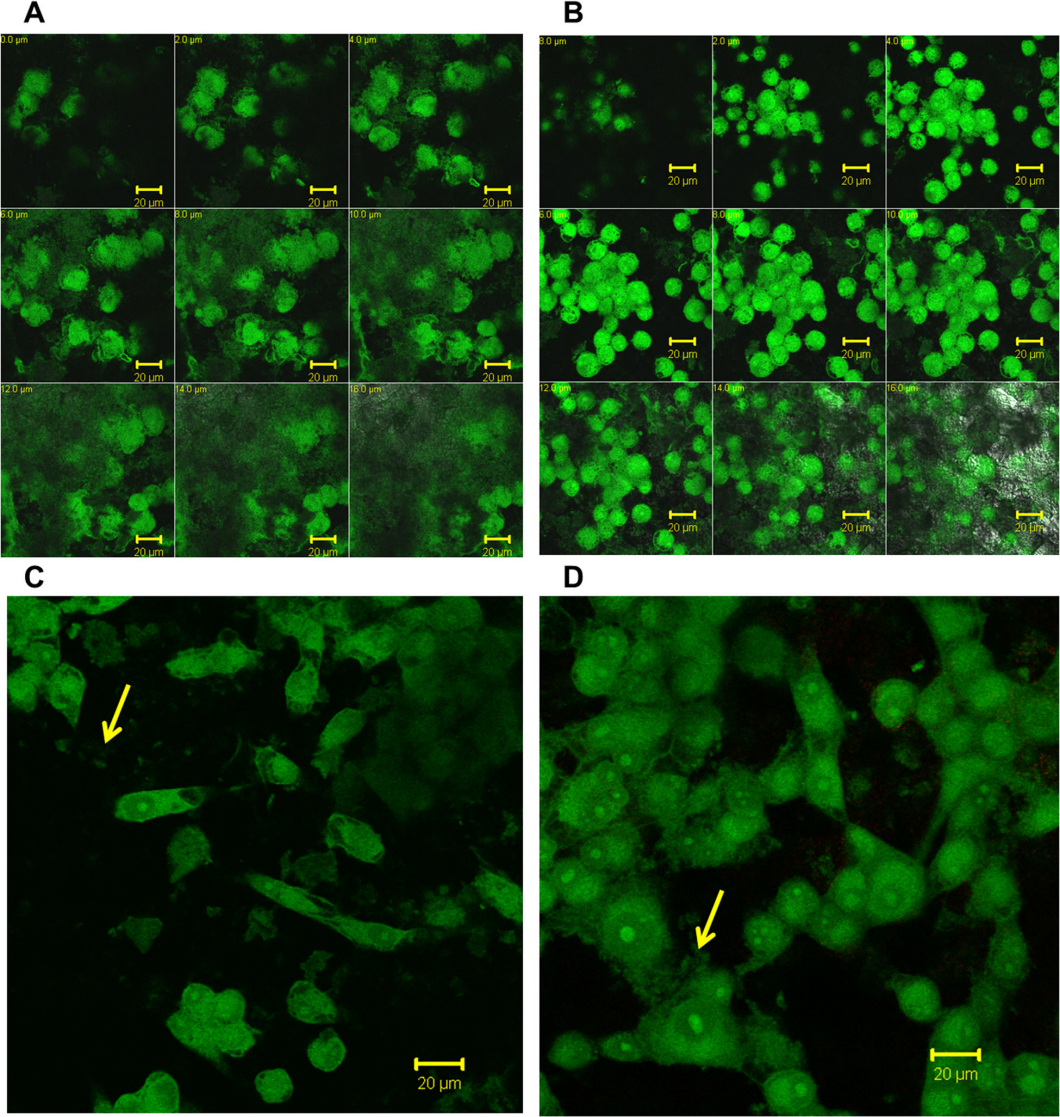
Representative z-stacked (Panels **A**, **B**) / representative single (Panels **C**, **D**) images for Osteocalcin expression in MG-63 cells cultured on coated Titanium substrate (Panel **A**, **C**: 7 days; Panel **B**, **D**: 14 days). Cultured Titanium samples were stained with anti human Osteocalcin and secondary antibody conjugated with FITC. Images were captured on Laser confocal microscope using emission filter BP 505–550. Scale Bar in microns is shown at bottom of each panel. Arrows in panels (**C**) and (**D**) indicate secreted osteocalcin

Coated titanium substrate was assessed for osteoblastic marker protein, osteocalcin to check whether the osteoblastic phenotype of the MG-63 cells was restored or altered following attachment on the substrate. Osteocalcin is one of the most abundant proteins produced by bone and is likely to be involved in bone remodeling through its interaction with other ECM molecules. The level of osteocalcin within MG-63 cells adhering on the coated titanium substrates was found be increased significantly from day 7 to 14 days (Fig. 5A, B, Supplementary Videos 2 and 3). The continuous expression of this osteoblastic marker indicates the maintenance of the osteoblastic phenotype by cells attached to the titanium substrates. Figure 5A, B show the z-stacked images over a depth of 20-micron and at 20-micron depth, the substrate is seen as a silvery layer. This indicates that cells occupy depth of 20 micron which is in line with size of cells between 10–20 micron.

## Discussion

Tissue compatibility of metallic biomaterials plays an important role in the success of implants. Titanium’s remarkable ability to osseointegrate makes it an attractive biomaterial for a variety of clinical applications viz. dental/maxillofacial implants and joint prostheses. Its use is showing increasing trend in artificial hip, knee replacements, as screw to hold prostheses or repair injuries, or implant materials including pacemakers and cochlear implants. Bone remodeling and mineralization processes at the bone-implant interface are fundamental elements for the long-term survival of implanted prostheses [17]. Macro, micro and nano topography of the surface and the hydrophilicity plays an important role in interaction of host osteoblasts with biomaterial surface during bone formation [18]. In the case of failing implants’ nonmineralized connective tissue forms at the interface of the implant leading to loss of osseointegration.

The present study evaluated the effect of nanocomposite coating consisting of ceramic AW reinforced with polymer chitosan deposited by electrophoretic deposition on osteoblast behavior on Ti substrates. HA is one of the most widely used forms of the most widely used forms of calcium phosphate ceramics [19]. The morphological structure of HA is similar to apatite phase of bone and HA coated implants have shown osteoconductive properties which improve early bone ingrowth and mechanical fixation of implants [20]. Long term results have brought out some concerns about HA. A clinical trial with 10 years follow up consisting of 191 patients using gritblasted HA coated acetabular cups confirmed concerns such as resorption and delamination of the coating as failure mechanisms, especially in younger patients [21]. In an in-vitro study by Łukaszewska-Kuska M, the electrochemical deposition of HA on endosseous implants showed uniform, crack-free and thin layer of HA with surface roughness potentially conducive to tissue reaction [22].

Bone cell culture models have been successfully employed to study bone-biomaterial interactions since several decades [23]. Various cell types such as mouse fibroblasts (L929, 208 F), human osteosarcoma cells (MG-63, SaOS-2), human lung carcinoma (A549), rat calvarial osteoblasts, osteoblasts from human trabecular bone have been routinely used for assessing biocompatibility of tissue engineering scaffolds [24]. These cultures provide morphological, biochemical and molecular information regarding osteoblastic development and synthesis of matrix at the interface with various biomaterials. The de-novo bone formation is a multistep cascade initiated with chemotaxis and attachment of osteogenic cells to the solid substratum [25]. The attached osteogenic cells undergo a temporal sequence of gene expression defined into distinct proliferation, differentiation and mineralization [26]. Hence it is important to understand influence of the biomaterial surface on cell attachment and proliferation.

In this study, the authors have used osteoblast-like cells, MG-63 to understand the biological effect of the Ti surface with the composite coating. It is well documented that osteocalcin is one of the bone specific proteins synthesized by osteoblasts, which is used as a marker of bone formation. Ivaska et al. have demonstrated that osteocalcin is released by osteoblasts during bone formation both as intact molecules and fragments [27]. The detection of osteocalcin in the culture medium provides a useful tool for monitoring bone turnover marker in addition to marker of bone formation [26].

Bellare JR and colleagues had previously demonstrated ability of this nanocomposite coating made of ceramic and biopolymer for titanium implants, to form bone-like matrix in acellular simulated body fluid [11]. Further they demonstrated using a rabbit tibial defect model, the ability of the composite coated Ti in bringing about extensive calcification of cartilage and formation of bone trabeculae at implantation site while at the uncoated Ti site mild calcification of cartilage and formation of bony trabeculae was observed [12]. In order to understand the mechanism underlying these observations, present in vitro studies were performed to compare ability of coated Ti to support osteoblast adhesion and function over uncoated Ti substrates.

Morphological differences were observed between the cells cultured on uncoated and coated titanium substrate. Our results showed that our composite coating was non-toxic to the MG-63 cells that were cultured on coated titanium substrates. Moreover, they adhered well with flattened morphology, proliferated and secreted ECM faster than uncoated titanium substrate. Adherence, growth and ECM secretion are must for osteogenesis to occur in the immediate vicinity of the implanted material in vivo. These findings indicated that the AW-Chitosan coating promoted cell attachment and proliferation. The cell attachment and proliferation observed on uncoated titanium could be due to a thin layer of native oxide layer formed on uncoated Ti substrate [28, 29]. However, this growth was significantly less than that supported by composite coating.

The synthesis of the bone-related protein osteocalcin is a phenotypic expression of the osteoblast like behavior. From the osteocalcin staining of the MG-63 cells grown on coated titanium substrates it can be inferred that the osteoblastic function and phenotype of the MG-63 cells were enhanced. The osteocalcin is secreted in extracellular compartment and then mineralizes to give mineralized matrix and calcium nodules which is a key factor for bone regeneration. For this purpose, it is possible to use a histochemical technique using alizarin red stain to detect calcium deposits. Intense alizarin red staining for the coated substrates as compared to the uncoated substrates indicated faster mineralization in presence of coating on Ti. The results of the present study are in agreement with earlier reports that expression of osteocalcin is coupled with deposition of hydroxyapatite and mineralization [26].

## Conclusion

Limitation of the study is that accuracy of supporting osteoblasts growth could be tested only upto 14 days, as it’s a pre-clinical in vitro study model. Further it does not involve cross-talk to regulate osteoblasts-implant surface interactions by other cellular/soluble components of extracellular matrix, in physiological microenvironment. However, we have successfully demonstrated that titanium substrates coated with AW-Chitosan by using electrophoretic deposition significantly enhanced the osteoblast cell viability, adhesion and proliferation as compared to uncoated titanium substrates. Significant increase in the expression of osteocalcin by the cells on coated substrates and increased in-vitro mineralization confirmed the biocompatibility of the AW-C coating and its ability to initiate early osseointegration of implants in contact with hard and soft tissue interfaces, thus confirming its applications in the field of oral craniofacial implantology, osseointegration based prostheses in amputee care and prosthetic hip and knee replacements.

## Supplementary Information

Supplementary Video 1

Supplementary Video 2

Supplementary Video 3

Ti supplementary Figures

Captions to cover images 120821

## Data Availability

The data that support the findings of this study are available from the corresponding author upon reasonable request.
