# Supplementary material for: Improved osteoblast function on titanium implant surfaces coated with nanocomposite Apatite–Wollastonite–Chitosan– an experimental in-vitro study
Source: J Mater Sci Mater Med. 2022 Feb 21;33(3):25. doi: 10.1007/s10856-022-06651-w (PMC8860945; doi:10.1007/s10856-022-06651-w)
Supplement: Supplementary file 4 — Ti supplementary Figures [file 10856_2022_6651_MOESM4_ESM.pdf]

Supplementary Material 1

A

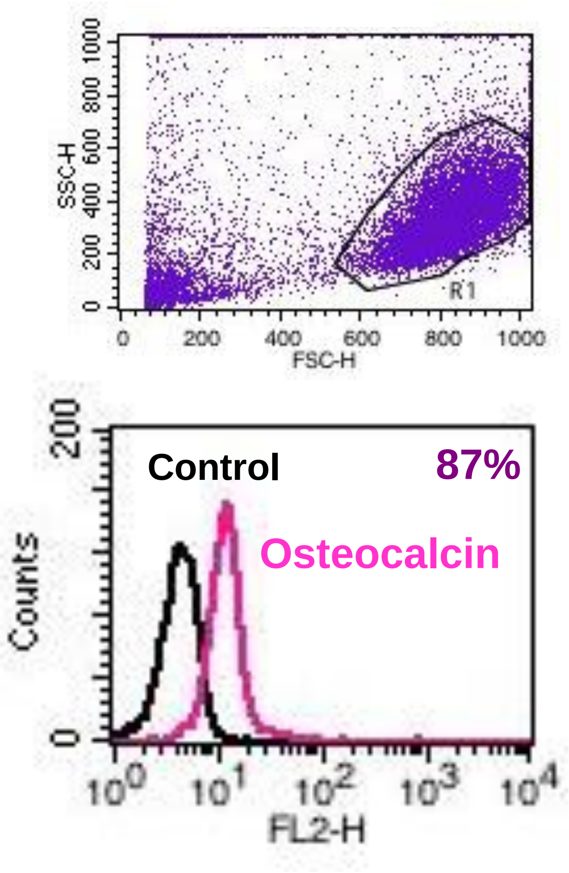

B

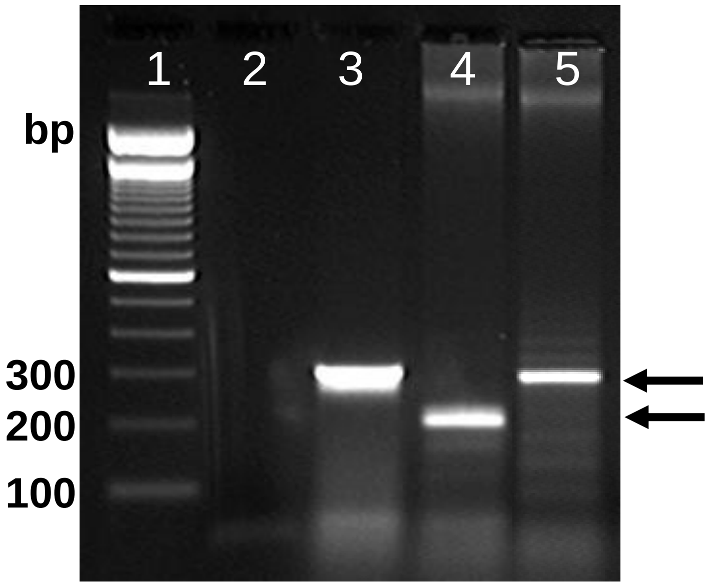

Supplementary Material 2

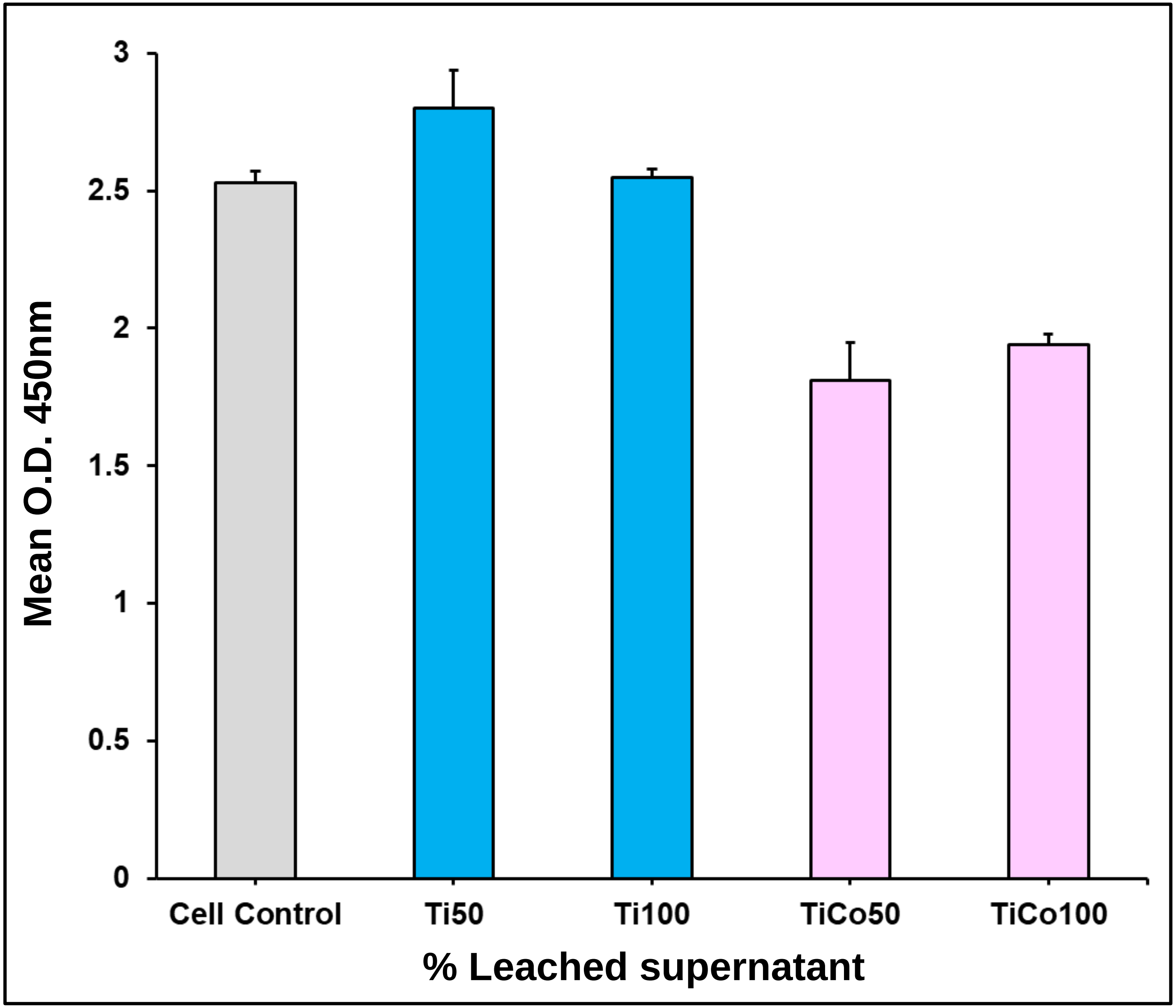

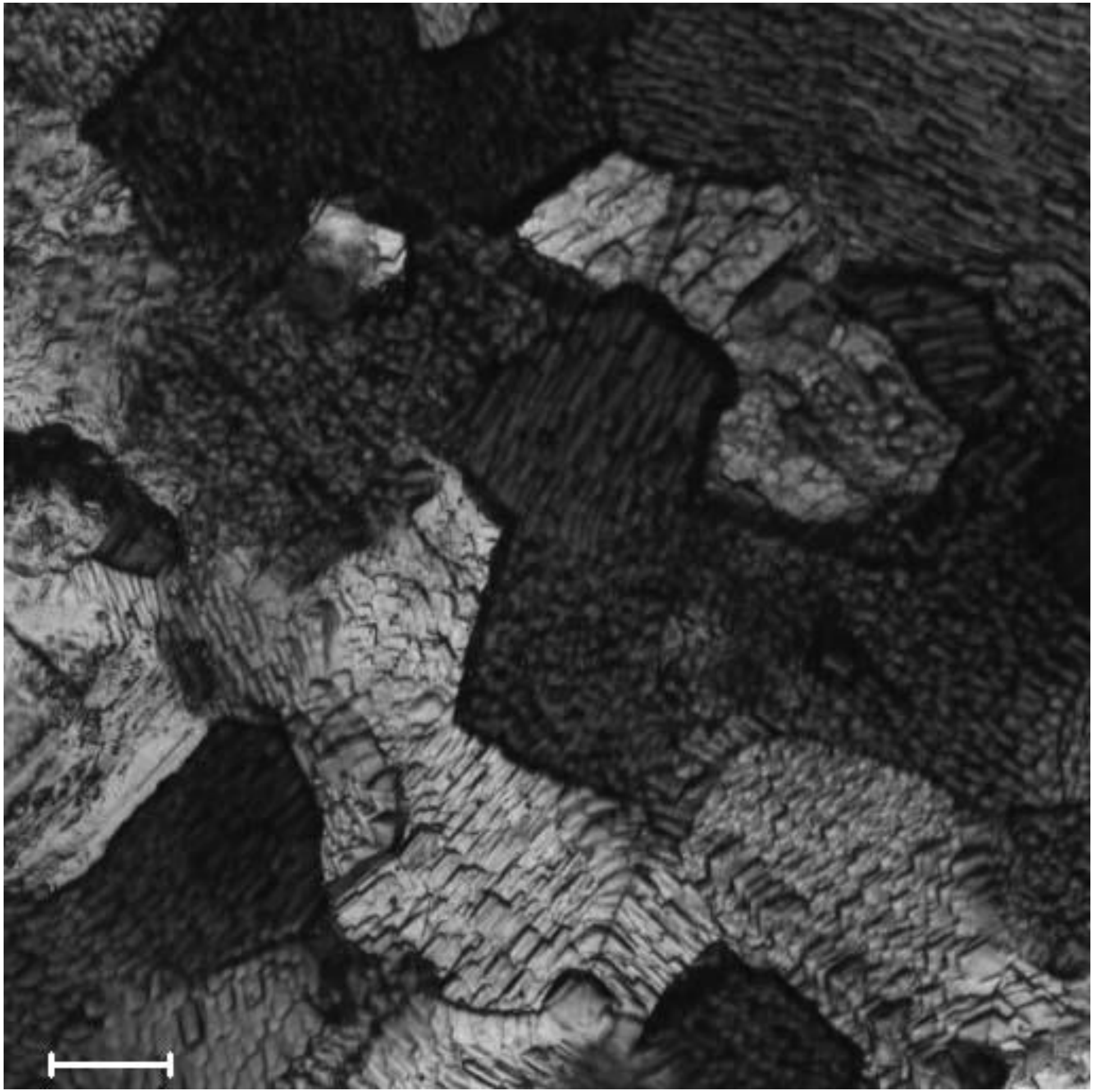

Supplementary Material 3

Scale Bar: 10 micron

Supplementary Material 4

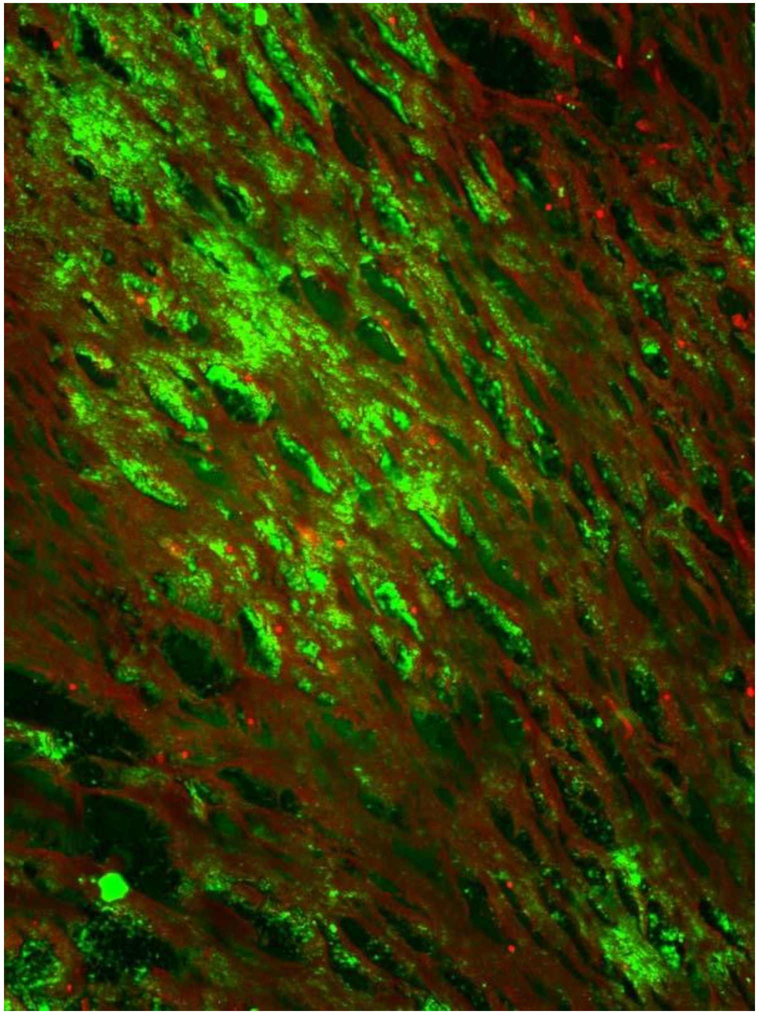

Trabecular bone : Red staining indicates mineralized calcium while green color indicates bone cellular region
