# Supplementary material for: Improved osteoblast function on titanium implant surfaces coated with nanocomposite Apatite–Wollastonite–Chitosan– an experimental in-vitro study
Source: J Mater Sci Mater Med. 2022 Feb 21;33(3):25. doi: 10.1007/s10856-022-06651-w (PMC8860945; doi:10.1007/s10856-022-06651-w)
Supplement: Supplementary file 5 — Captions to cover images 120821 [file 10856_2022_6651_MOESM5_ESM.docx]

**Cover Image -1**


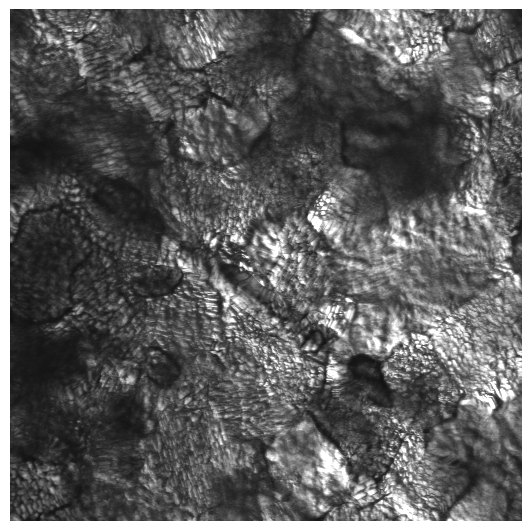


Caption: Transmission confocal microscopy image of titanium implant coated with nanocomposite **Apatite-Wollastonite-Chitosan** with MG-63 osteoblasts cells grown for 14 day on its surface

**Cover Image -2**


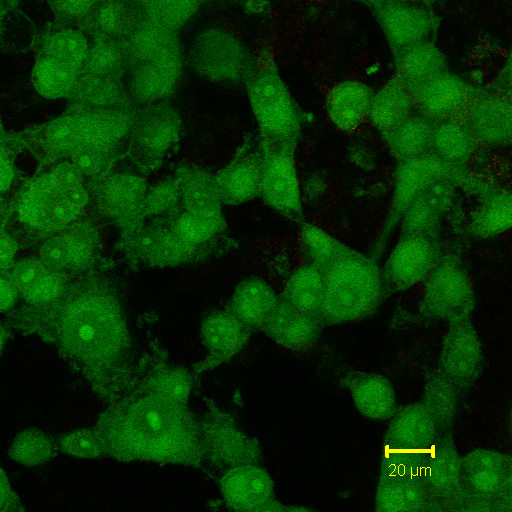


Caption: Osteocalcin staining image by confocal microscopy of titanium implant coated with nanocomposite **Apatite-Wollastonite-Chitosan** with MG-63 osteoblasts cells grown for 14 day on its surface

**Cover Image -3**


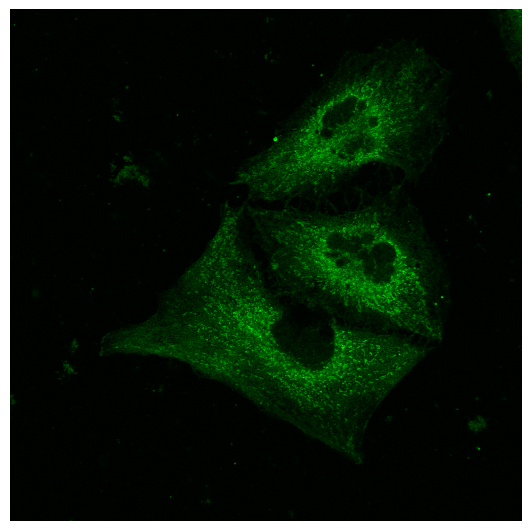


Caption: Osteocalcin staining image by confocal microscopy of MG-63 osteoblasts cells. Green fluorescence indicates intracellular as well as secreted osteocalcin which later mineralizes to forms bone.

**Cover Image -4**


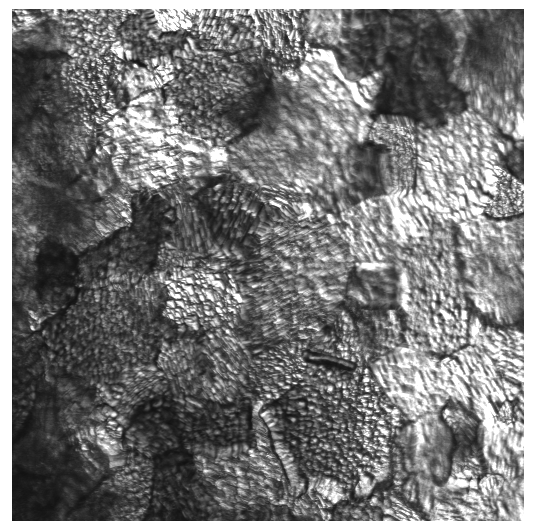


Caption: Transmission confocal microscopy image of titanium implant coated with nanocomposite **Apatite-Wollastonite-Chitosan** with MG-63 osteoblasts cells grown for 14 day on its surface

**Cover Image -5**


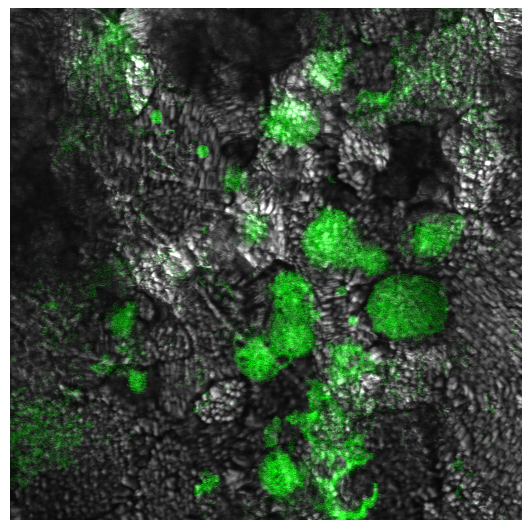


Caption: Confocal microscopy image of titanium implant coated with nanocomposite **Apatite-Wollastonite-Chitosan** with MG-63 osteoblasts cells grown for 14 day on its surface. Dual of Green fluorescence and Transmission image where, green fluorescence indicates intracellular and secreted osteocalcin expression by MG-63 cells.
